# Cardiac troponin for predicting all-cause mortality in patients with acute ischemic stroke: a meta-analysis

**DOI:** 10.1042/BSR20171178

**Published:** 2018-03-09

**Authors:** Yu Fan, Menglin Jiang, Dandan Gong, Changfeng Man, Yuehua Chen

**Affiliations:** 1 Institute of Molecular Biology and Translational Medicine, The Affiliated People’s Hospital, Jiangsu University, Zhenjiang, Jiangsu 212002, P.R. China; 2 Department of Nuclear Medicine, Affiliated Hospital of Jiangsu University, Zhenjiang, Jiangsu 212001, P.R. China

**Keywords:** acute ischemic stroke, all-cause mortality, cardiac troponin, meta-analysis

## Abstract

Cardiac troponins are specific biomarkers of cardiac injury. However, the prognostic usefulness of cardiac troponin in patients with acute ischemic stroke is still controversial. The objective of this meta-analysis was to investigate the association of cardiac troponin elevation with all-cause mortality in patients with acute ischemic stroke. PubMed and Embase databases were searched for relevant studies up to April 31, 2017. All observational studies reporting an association of baseline cardiac troponin-T (cTnT) or troponin-I (cTnI) elevation with all-cause mortality risk in patients with acute ischemic stroke were included. Pooled adjusted risk ratio (RR) and corresponding 95% confidence interval (CI) were obtained using a random effect model. Twelve studies involving 7905 acute ischemic stroke patients met our inclusion criteria. From the overall pooled analysis, patients with elevated cardiac troponin were significantly associated with increased risk of all-cause mortality (RR: 2.53; 95% CI: 1.83–3.50). The prognostic value of cardiac troponin elevation on all-cause mortality risk was stronger (RR: 3.54; 95% CI: 2.09–5.98) during in-hospital stay. Further stratified analysis showed elevated cTnT (RR: 2.36; 95% CI: 1.47–3.77) and cTnI (RR: 2.79; 95% CI: 1.68–4.64) level conferred the similar prognostic value of all-cause mortality. Acute ischemic stroke patients with elevated cTnT or cTnI at baseline independently predicted an increased risk of all-cause mortality. Determination of cardiac troponin on admission may aid in the early death risk stratification in these patients.

## Introduction

Acute ischemic stroke is caused by permanent brain injury secondary to disruption of blood flow [1]. Approximately 68% of strokes are classified into ischemic stroke [2]. From 2000 to 2010, age-specific acute ischemic stroke hospitalization rates increased for individuals aged 25–64 years in the United States [3]. Ischemic stroke remains the leading cause of disability and second cause of deaths throughout the world [4].Cardiac complications account for the leading cause of mortality in these patients [5]. Therefore, determining the prognosis after acute ischemic stroke is an unmet need.

Cardiac troponin-T (cTnT) and cardiac troponin-I (cTnI) are biochemical markers of cardiac injury [6]. An increase in the serum level of cTnT or cTnI is common in the acute phase of ischemic stroke [7], indicating a close relationship between stroke and cardiac damage. It should be noted that increase in cardiac troponin in acute stroke may be explained by comorbidities in some cases [8]. However, inconsistent results [8–21] have been yielded on the association of elevated cardiac troponin level with all-cause mortality risk in patients with acute ischemic stroke. Moreover, the strength of risk estimates varied significantly across studies. Previously published systematic review [22] did not establish the conclusion that troponin elevation was an independent prognostic factor in acute ischemic stroke patients. So far, prognostic value of cardiac troponin elevation in stroke patients remains conflicting.

The purpose of this meta-analysis was to evaluate the association of baseline cTnT or cTnI elevation with all-cause mortality risk in patients with acute ischemic stroke based on available observational studies.

## Materials and methods

### Data source and literature search

We conducted this meta-analysis according to the reporting checklist of the Meta-analysis of Observational Studies in Epidemiology [23]. To identify relevant studies, an electronic literature search was conducted in PubMed and Embase databases from their inception to April 2017. The terms searched were a combination of the following keywords: ‘troponin’ AND ‘stroke’ OR ‘cerebrovascular accident’ AND ‘mortality’ OR ‘death’. Included studies were restricted in an English publication. In addition, we also manually searched the bibliographies of all the relevant articles to identify additional studies.

### Study selection

Studies included in this meta-analysis should satisfy the following inclusion criteria: (i) participants: patients with acute ischemic stroke; (ii) study design: prospective or retrospective observational studies; (iii) exposure: at least one determination of serum level of cTnT or cTnI on admission; (iv) outcome measure: follow-up all-cause mortality or in-hospital death; and (v) provided multivariate-adjusted risk estimate of all-cause or in-hospital mortality associated with the elevated serum cardiac troponin category. Studies were excluded if: (i) participants included hemorrhagic stroke patients; (ii) not providing mortality outcomes based on troponin values; and (iii) reported unadjusted risk estimate.

### Data extraction and quality assessment

From each of the eligible studies, two authors independently extracted the following data: surname of the first author, publication year, region of study performed, study design, sample size, proportion of men, mean age or age range, type of cardiac troponin, cut-off value of cardiac troponin, percentage of abnormal cardiac troponin, follow-up time, number of deaths, maximum adjusted risk estimate, and adjustment for covariates. Any discrepancies between two authors were resolved in the presence of a third reviewer. To perform quality assessment, a maximal 9-star Newcastle–Ottawa Scale (NOS) of cohort studies [24] was applied to assess the methodological quality of each study. According to the NOS score, study awarding 7 or over stars was considered good quality.

### Statistical analysis

Meta-analyses were carried out with STATA (version 12.0, Stata, College Station, TX, U.S.A.). Summary risk ratio (RR) with 95% confidence interval (CI) was pooled for patients with elevated serum cardiac troponin compared with undetectable cardiac troponin category. Statistical heterogeneity between studies was evaluated by the *I^2^* statistic and Cochran’s Q test. The *I^2^* > 50% or *P*<0.10 for Cochran’s Q test was regarded as presence of statistical heterogeneity. In presence of significant heterogeneity, a random effect model was used; otherwise, we selected a fixed-effect model. Subgroup analyses were performed by the type of cardiac troponin measured (cTnT or cTnI), assay of troponin (conventional or high-sensitivity), study design (prospective or retrospective), length of follow-up (in-hospital or follow-up), whether excluded coronary heart disease (CHD)/acute myocardial infarction (AMI) patients (yes or no), whether adjustment for renal dysfunction (yes or no), and NOS (≥7 stars compared with <7 stars). Publication bias was explored by the Egger’s linear regression test [25]. Sensitivity analyses were conducted by excluding any one study at each time to observe the influence of a single study on the pooled summary.

## Results

### Search results and study characteristics

**Figure 1:**
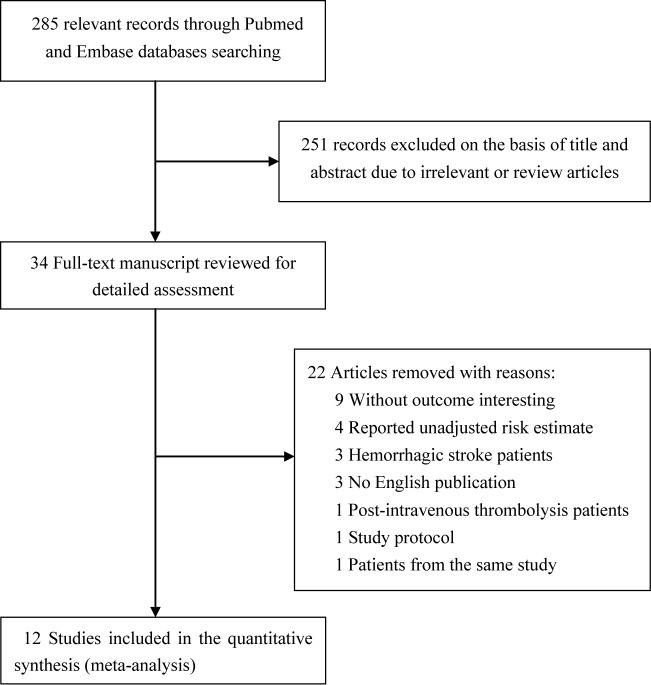
Flow diagram of the study selection process

Figure 1 shows the flow diagram for the study selection process. Briefly, a total of 285 records were obtained from the initial electronic search. After screening titles and abstracts, 251 articles were excluded because they were obviously irrelevant or review articles. Two articles [8,26] conducted by Faiz et al. selected the same participants; however, they reported the in-hospital death and follow-up mortality, respectively. After thorough evaluation of the full-text manuscript, 12 studies [8,10–16,18–21] were finally included in this meta-analysis. Table 1 summarizes the baseline clinical and demographic characteristics of the included studies. A total of 7905 acute ischemic stroke patients were included. The sample size of the selected studies ranged from 106 to 1718. Seven studies [10–12,14–16,20] were prospective, whereas five [8,13,18,19,21] were retrospective in nature. Eight studies [8,10–16] were conducted in Europe, two [19,20] in the United States, and two [18,21] in Asia. The percentage of patients with detectable cardiac troponin varied from 10 to 60%. Studied duration ranged from in-hospital stay to 4.4 years. Using the NOS scale, the selected studies were classified into moderate to good quality (Table 3).

**Table 1:** Summary of characteristics of the included studies

Author (year)	Region	Study design	Patients (% men)	Age (years)	cTn type/cut-off values	Abnormal cTn (%)	Event number OR/HR (95% CI)	Follow-up duration	Maximum adjusted covariates
Di Angelantonio et al. (2005) [10]	Italy	Prospective study	330 (51.5)	57.6 ± 12.9	cTnI; 0.1 ng/ml	16.3%	Total deaths: 65; 2.28 (1.42–3.67)^*^	144 months	Age and baseline NIHSS score
Jensen et al. (2007) [11]	Denmark	Prospective study	244 (52.5)	68.7 ± 13.1	cTnT; 0.03 μg/l	10%	Total deaths: 36; 3.39 (1.34–8.60)	19 months	Age, Scandinavian Stroke Scale score, and heart and/or renal failure
Jensen et al. (2012) [12]	Denmark	Prospective study	193 (56.5)	69.4 ± 12.1	Hs–cTnT; 14 ng/l	33.7%	Total deaths: 43; 1.32 (0.62–2.81)	4.4 years	Age, C-reactive protein, NT-proBNP, prior heart and/or renal failure, and stroke severity
Scheitz et al. (2012) [13]	Germany	Retrospective study	715 (49.8)	66–84	cTnT; 0.03 μg/l	14%	In-hospital deaths: 26; 4.51 (1.93–10.57)	—	Age and stroke severity
Hajdinjak et al. (2012) [14]	Slovenia	Prospective cohort study	106 (58.5)	70.0 ± 12.1	cTnT; 0.04 μg/l	15.1%	In-hospital deaths: 23; 1.8 (1.1–8.4)	—	Age, SBP, DBP, NIHSS score, NT-proBNP, and blood glucose
Scheitz et al. (2014) [15]	Germany	Prospective study	1016 (49.1)	61–88	Hs–cTnT; 14 ng/l	60%	In-hospital deaths: 36; 1.81 (0.80–4.10)	—	Age, gender, prestroke independence, NIHSS score, AF, congestive heart failure, and insular cortex involvement
Faiz et al. (2014) [8]	Norway	Retrospective study	287 (55.1)	65–83	Hs–cTnT; 14 ng/l	54.4%	In-hospital deaths: 17 1.15 (0.17–4.22); total deaths: 88^∧^; 1.65 (1.04–2.63)	1.5 years	Age, gender, NIHSS, CHD, cerebrovascular disease, AF, smoking, hypertension, DM, and eGFR
Lasek-Bal et al. (2014) [16]	Poland	Prospective study	1068 (57.0)	72 ± 11	Hs–cTnI; 0.014 ng/ml	9.7%	Total deaths:142 3.05 (1.65–5.65)	1 month	Age, NIHSS, hypertension, DM, lipid, AF, and CHD
Maoz et al. (2015) [18]	Israel	Retrospective study	212 (56.1)	73.9 ± 12.9	Hs–cTnT; 0.03 μg/l	16.5%	In-hospital deaths: 23; 22.57 (4.4–116.6)	—	Age, ischemic heart disease, creatinine, creatinine clearance, and NIHSS score
Peddada et al. (2016) [19]	U.S.A.	Retrospective study	1145 (55.1)	65 ± 15	Hs–cTnI; 0.12 ng/ml	17.0%	In-hospital deaths: 129; 4.28 (2.40–7.63)	—	Age, gender, smoking, AF, heart failure, hemiplegia, dysphagia, hemorrhagic complication, respiratory failure, renal dysfunction, creatinine, use of oral antiplatelet ot systemic anticoagulation therapy
Batal et al. (2016) [20]	U.S.A.	Prospective study	1718 (50)	67 ± 15	cTnI; 0.10 μg/l	18%	Total deaths: 413; 1.44 (1.10–1.89)	1.4 years	Age, comorbid factors, stroke etiology, admission SBP, NIHSS score, and creatinine
Su et al. (2016) [21]	Taiwan	Retrospective study	871 (NP)	72.3 ± 13.6	cTnI; 0.01 μg/l	16.8%	In-hospital deaths: 31 5.59 (2.36–13.27)	—	Gender, evidence of clinical deterioration, and NIHSS score

Abbreviations: AF, atrial fibrillation; BMI, body mass index; CK, creatine kinase; COPD, chronic obstructive pulmonary disease; DBP, diastolic blood pressure; DM, diabetes mellitus; eGFR, estimated glomerular filtration rate; HR, hazard ratio; Hs, high-sensitivity; NP, not provided; NIHSS, National Institutes of Health Stroke Scale; SBP, systolic blood pressure. *Combined from each category of troponin I. ^†^Data from Faiz et al. (2014) [26].

**Table 2:** Quality assessment of studies included in meta-analysis

Study (year)	Representativeness of the exposed cohort	Selection of the non-exposed cohort	Ascertainment of exposure	Demonstration that outcome was not present at the start of study	Comparability of cohorts on the basis of the design or analysis	Assessment of outcome	Enough follow-up periods (≥1 year)	Adequacy of follow-up of cohorts	Overall NOS
Di Angelantonio et al. (2005) [10]	★	★	★	★	★	★	★	★	8
Jensen et al. (2007) [11]	★	★	★	★	★	★	★		7
Jensen et al. (2012) [12]	★	★	★	★	★	★	★		7
Scheitz et al. (2012) [13]	★	★	★	★	★	★	★		7
Hajdinjak et al. (2012) [14]	★	★	★	★	★	★			6
Scheitz et al. 2014 [15]	★	★	★	★	★	★			6
Faiz et al. (2014) [8]	★	★	★	★	★	★			6
Lasek-Bal et al. (2014) [16]	★	★	★	★	★	★		★	7
Maoz et al. (2015) [18]	★	★	★	★	★	★			6
Peddada et al. (2016) [19]	★	★	★	★	★	★			6
Batal et al. (2016) [20]	★	★	★	★	★	★	★		7
Su et al. (2016) [21]	★	★	★	★	★	★			6

★ denotes a result that satisfies the requirement of the column label.

**Table 3:** Subgroup analyses on all-cause mortality

Subgroup	Number of studies	Pooled RR	95% CI	Heterogeneity between studies
Study design				
Prospective	7	1.92	1.46–2.53	*P*=0.186; *I^2^* = 31.7%
Retrospective	5	3.75	1.97–7.17	*P*=0.186; *I^2^* = 31.7%
Region				
European	8	2.16	1.68–2.77	*P*=0.354; *I^2^*= 9.7%
Non-European	4	4.33	1.63–11.45	*P*<0.001; *I^2^* = 88.5%
Type of troponin				
cTnT	7	2.36	1.47–3.77	*P*=0.031; *I^2^*= 54.7%;
cTnI	5	2.79	1.68–4.64	*P*=0.001; *I^2^*= 79.7%
Assay of troponin				
Conventional	6	2.13	1.41–3.20	*P*=0.027; *I^2^* = 60.3%;
High-sensitivity	6	3.00	1.83–4.92	*P*=0.010; *I^2^* = 64.2%
Excluded CHD/AMI patients				
Yes	7	2.74	1.96–3.84	*P*=0.061; *I^2^* = 48.0%
No	5	2.31	1.21–4.41	*P*=0.009; *I^2^* = 70.2%
Adjusted renal function				
Yes	7	2.25	1.43–3.53	*P*=0.002; *I^2^* = 69.9%
No	5	2.95	2.05–4.25	*P*=0.236; *I^2^* = 27.8%
NOS				
≥7 stars	6	2.45	1.55–3.89	*P*=0.005; *I^2^* = 70.5%
<7 stars	6	3.00	1.73–5.21	*P*=0.050; *I^2^*= 54.8%

### All-cause mortality

**Figure 2:**
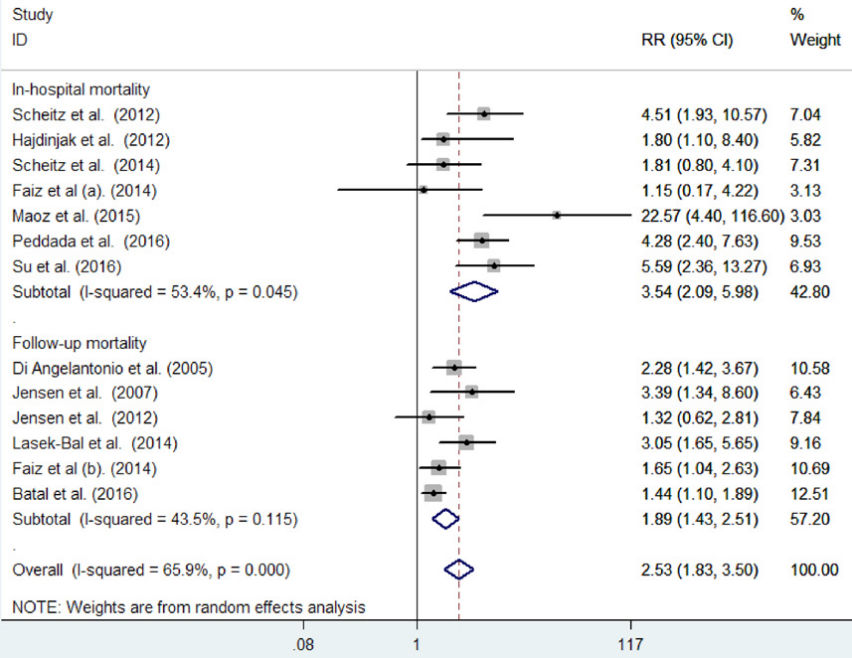
Forest plots showing the association of baseline cardiac troponin elevation with all-cause mortality in patients with acute ischemic stroke

All included studies reported the effect of cardiac troponin elevation on the risk of all-cause mortality in-hospital stay [8,13–15,18,19,21] and at the end of follow-up duration [10–12,16,20]. As shown in Figure 2, significant heterogeneity was detected (*I^2^* = 65.9%; *P*<0.001) across the studies. Meta-analysis using a random effect model showed that acute ischemic stroke patients with elevated cardiac troponin level significantly increased risk of all-cause mortality (RR: 2.53; 95% CI: 1.83–3.50). Further, pooled RR was 3.54 (95% CI: 2.09–5.98) and 1.89 (95% CI: 1.43–2.51) for in-hospital mortality and follow-up mortality, respectively. Sensitivity analysis by removing a single study by turns indicated that there was no obvious impact of any individual study on the pooled risk estimate (data not shown).

### Subgroup analyses and publication bias

Subgroup analyses suggested that patients with acute ischemic stroke had a higher risk of all-cause mortality in the subgroup of retrospective study, non-European, use of high-sensitivity troponin assays, unadjusted renal function, included CHD, and NOS scores <7 stars (Table 2). Possible publication bias was observed in the overall analysis of all-cause mortality (*P*=0.046 for the Egger’s test). However, no publication bias was detected according to the results of Egger’s test, *P*=0.993 in-hospital mortality and *P*=0.156 in follow-up mortality.

## Discussion

A total of 12 eligible studies with total 7905 acute ischemic stroke patients were identified and analyzed in this meta-analysis. Overall, these selected studies had a moderate-to-good methodological quality. The principal finding of this meta-analysis suggested that acute ischemic stroke patients with cTnT or cTnI elevation were associated with an increased risk for all-cause mortality. Acute ischemic stroke patients with cardiac troponin elevation had a 2.54-times and 89% higher risk of in-hospital deaths and follow-up all-cause mortality, respectively.

The etiology of troponin elevation in acute stroke is not fully understood [27]. Cardiac troponin elevation in the context of acute stroke is associated with the presence of chronic conditions, such as congestive heart failure, impaired renal function [8,13]. Other causes of detectable cardiac troponin include: chronic obstructive pulmonary disease, pulmonary embolism, sepsis, chronic renal failure, and atrial fibrillation [28]. Silent myocardial infarction could have contributed to the poor prognosis of these patients. However, the effects of cardiac troponin elevation on all-cause mortality risk were still statistically significant in pooled studies excluding patients with AMI or pre-existing CHD. Also, elevated cardiac troponin significantly predicted the risk of all-cause mortality despite adjustment for the presence of renal dysfunction. These findings revealed that the prognostic value of abnormal cardiac troponin on mortality was independent of AMI and renal dysfunction. Therefore, troponin elevation in these patients did not seem to be solely a consequence of AMI or renal dysfunction.

Subgroup analysis showed a slight discrepancy in prognostic value between cTnT or cTnI assays. Likewise, similarly prognostic value of cTnI and cTnT has been well established in acute coronary syndrome [29]. In addition, a stronger all-cause mortality risk was found in the subgroup of the retrospective study, in-hospital stay, non-European, and use of high-sensitivity troponin assays. The stronger effect in-hospital mortality may be partly explained by the coincidence of AMI [30].

Increase in troponin was associated with higher mortality in all types of stroke [12,31]. Initial cTnT elevation was a strong predictor of poor outcomes during the acute phase and long-term follow-up [32], even in the absence of acute coronary syndrome [33]. Cardiac troponin elevation also predicted the mortality in patients with intracerebral hemorrhage [34–36]. In spontaneous subarachnoid hemorrhage patients, a well-designed meta-analysis [37] has summarized that cardiac troponin elevation was associated with an increased risk of delayed cerebral ischemia and death.

One possible explanation of troponin elevation in acute ischemic stroke patients may be the coincidence of acute coronary syndrome, triggering a rise in circulating troponin level [38]. Cardiac vulnerability to cerebrogenic stress can be an alternate explanation interpretation of troponin elevation [39,40]. Activation of the sympathoadrenal system may be an important contributor to myocardial damage in acute ischemic stroke patients [41].

This meta-analysis is of clinical relevance to determine the prognostic value of cTnT or cTnI in acute ischemic stroke patients. Given the frequency of abnormal troponin level in the period of acute ischemic stroke patients, routinely determine the level of cardiac troponin in these patients may help identify patients who faced higher risk of post-stroke death. In cases with cardiac troponin increase, one should identify comorbidities associated with cardiac troponin elevation.

Several shortcomings in this meta-analysis should be noted. First, single troponin measurement at baseline may have led to misclassification of patients in each category. Repeated measurements over time can provide more accurate information on the myocardial injury in patients with acute ischemic stroke. Second, lack of adjustment for residual confounding variables may have overestimated the risk estimate of the association between cardiac troponin elevation and mortality. Third, there was a substantial heterogeneity in polling risk summary, mainly due to differences in troponin assays or cut-off values, follow-up duration, and study design. Fourth, potential publication bias could not be excluded because the included studies were limited to English language publications. Finally, the prognostic value of elevated troponin level may be different in various stages or subtypes of ischemic stroke; therefore, the current findings could not generalize all stroke patients.

In conclusion, acute ischemic stroke patients with an elevated baseline cTnT or cTnI level were independently associated with an increased risk of all-cause mortality. Determination of cardiac troponin level should be considered for risk stratification in acute ischemic stroke patients. However, we could not recommend routine troponin measurement on all ischemic stroke patients due to the methodological flaws of the included studies. Further prospective studies are needed to address the prognosis in patients with acute ischemic stroke and concurrent elevated cardiac troponin.

## Abbreviations

AMI: acute myocardial infarction
CHD: coronary heart disease
CI: confidence interval
cTnI: cardiac troponin-I
cTnT: cardiac troponin-T
NOS: Newcastle–Ottawa Scale
RR: risk ratio
